# Benthic jellyfish act as suction pumps to facilitate release of interstitial porewater

**DOI:** 10.1038/s41598-023-30101-4

**Published:** 2023-03-07

**Authors:** David M. Durieux, Gabrielle D. Scrogham, Christian Fender, David B. Lewis, Stephen M. Deban, Brad J. Gemmell

**Affiliations:** University of South Florida, 4202 E Fowler Ave, Tampa, FL 33620 USA

**Keywords:** Biogeochemistry, Behavioural ecology, Biogeochemistry, Marine biology, Fluid dynamics

## Abstract

Upside-down jellyfish, genus *Cassiopea* (Péron and Lesueur, 1809), are found in shallow coastal habitats in tropical and subtropical regions circumglobally. These animals have previously been demonstrated to produce flow both in the water column as a feeding current, and in the interstitial porewater, where they liberate porewater at rates averaging 2.46 mL h^−1^. Since porewater in *Cassiopea* habitat can be nutrient-rich, this is a potential source of nutrient enrichment in these ecosystems. This study experimentally determines that porewater release by *Cassiopea* sp. jellyfish is due to suction pumping, and not the Bernoulli effect. This suggests porewater release is directly coupled to bell pulsation rate, and unlike vertical jet flux, should be unaffected by population density. In addition, we show that bell pulsation rate is positively correlated with temperature, and negatively correlated with animal size. As such, we would predict an increase in the release of nutrient-rich porewater during the warm summer months. Furthermore, we show that, at our field site in Lido Key, Florida, at the northernmost limit of *Cassiopea* range, population densities decline during the winter, increasing seasonal differences in porewater release.

## Introduction

**Figure 1:**
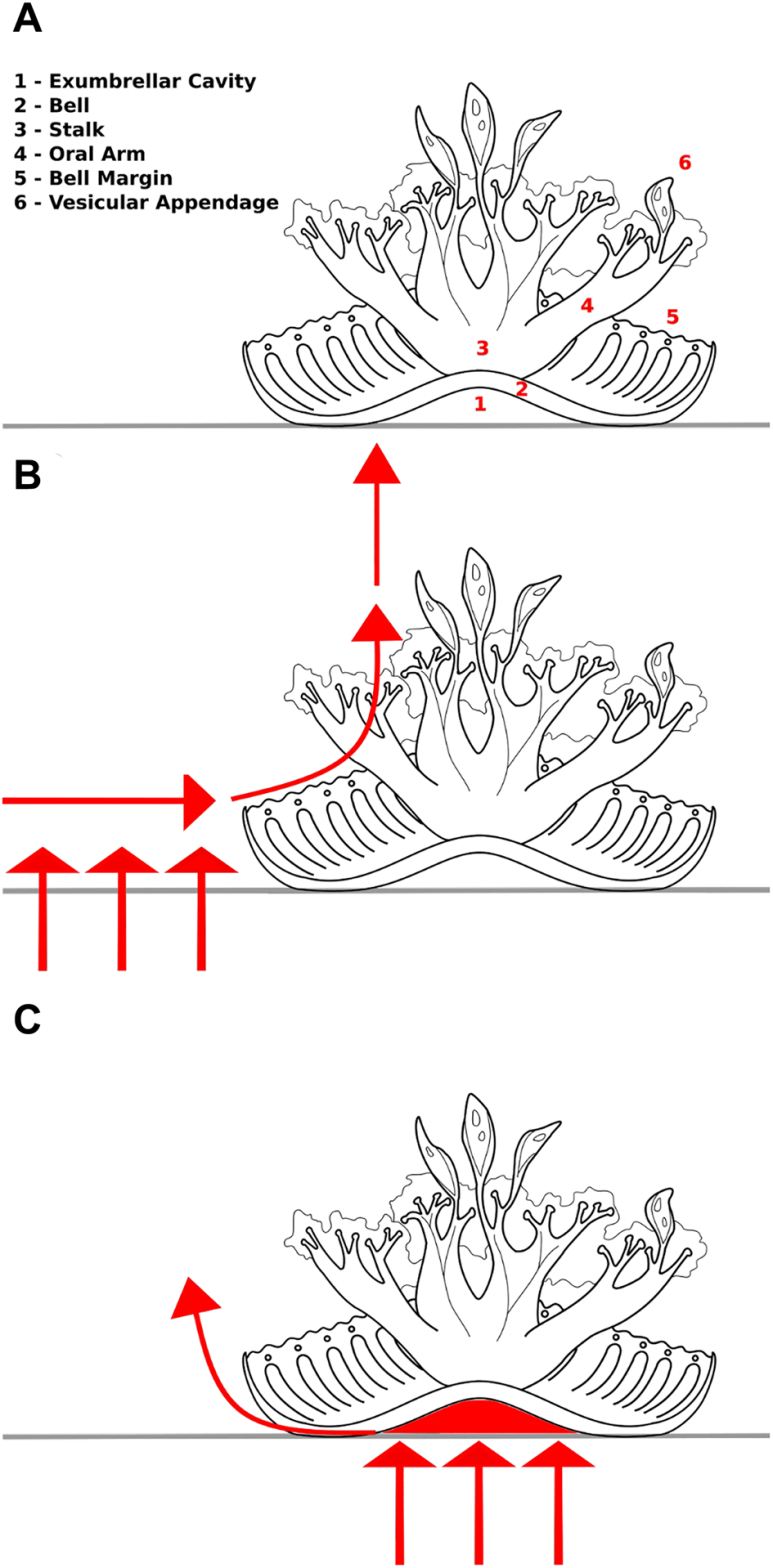
Hypotheses. Possible mechanisms for porewater transport by Cassiopea. Upside-down jellyfish are typically found on the benthos with their aboral side against the substrate and their oral surface facing upward. (**A**) The relevant anatomy of Cassiopea sp. includes the exumbrellar cavity, the water-filled space between the bell below the stalk and the benthos. (**B**) One of the tested hypotheses was that porewater would be liberated from the surrounding benthos via Bernoulli’s principle, below the region of relatively high-velocity water flow in the animal’s feeding current. (**C**) The other hypothesis under investigation was the “suction pump” mechanism, in which pressure fluctuations in the exumbrellar cavity draw porewater upwards below the bell, which is then released laterally as the bell moves in the process of bell pulsation.

As filter feeding animals, *Cassiopea* sp. (Péron and Lesueur, 1809) uses regular pulsation of their bells, *Cassiopea* sp. to draw water towards themselves horizontally along the substrate surface, pulling it into the bell below the oral arms^1^. As the water containing prey flows radially inward, it is redirected upwards through the oral arms, which are covered by nematocyst-laden digitata which capture prey items^2^. The filtered water is then ejected in the form of a vertical jet at velocities averaging between 1 and 2 cm s^−1^^3^, away from both the animal and the feeding zone (Fig. 1A)^1^. This process transports on the order of 200 L h^−1^ ind^−1^^3^.

In addition to transporting water through their feeding currents, *Cassiopea* sp. are also known to release benthic porewater into the water column as they pulse their bells^4,5^. The flux resulting from bell pulsations has been demonstrated to be 2.64 mL h^−1^ animal^−1^ for average-sized animals, scaling linearly with bell diameter^3^. Since *Cassiopea* sp. habitats are rich in nutrients^6^, *Cassiopea* sp. driven porewater release has been estimated to contribute an increase of as much as 29% to water column NH_4_ availability^3^, although a study on the long-term effects of this release has, to our knowledge, not yet been performed. In terms of flow velocity, porewater can be released from as deep as two cm within an hour of the addition of *Cassiopea* sp., indicating a minimum vertical flow velocity of 2 cm h^−1^^5^. This is significantly higher than rates reported in mangrove swamps lacking *Cassiopea* sp., which reported ranges of 1.1 cm day^−1^ in fast-flowing tidal creeks (which typically are not home to *Cassiopea* sp.) to 0.04 cm day^−1^ in mangrove habitat^7^.

While the physical transport of benthic porewater into the water column may have important ecological implications in areas where these animals are abundant, the underlying mechanism causing the liberation of porewater is unknown. Two hypotheses have been presented in the literature, developed based on two different aspects of the *Cassiopea* bell pulsing behavior. In the first hypothesis (Fig. 1B), incurrent flow travels horizontally along the bottom towards the animal, creating a low-pressure zone around the bell which entrains porewater^4^. As the water is accelerated along the substrate by the feeding current, pressure drops accordingly, creating a pressure gradient between the substrate surface and the water above by Bernoulli's principle. This would pull benthic porewater water from the substrate surrounding the bellPorewater is thus released and then entrained into the feeding current directly adjacent to the bell margin^4^.

The second hypothesis sees the exumbrellar cavity, the space between the bell of *Cassiopea* sp. and the substrate surface, acting as a suction pump when it deforms due to bell contraction^5^. This hypothesis treats *Cassiopea* sp. as a suction pump (Fig. 1C). In this case, the power stroke of the bell contraction horizontally expels the water captured under the concave bell, and restoration of bell shape during the recovery stroke produces a decrease in pressure under the bell that draws porewater up out of the sediment^5^. It was estimated that this mechanism could transport between 0.4 and 3.7 L m^−2^ h^−1^ and demonstrated, qualitatively, that porewater reaches the surface directly below the bell of *Cassiopea* sp.^5^.

It is the purpose of this study to determine to what extent these two mechanisms contribute to interstitial porewater release. This distinction is important to understanding the ecological role of *Cassiopea* sp., as increased porewater liberation due to horizontal flow can also be induced by tidal currents, potentially implying a lower impact of *Cassiopea* sp. relative to abiotic factors, while suction pumping would transport porewater in a manner unique to *Cassiopea* sp. In addition, if porewater is released from the region surrounding the animal through Bernoulli’s principle, then porewater release could be impeded by neighboring animals, leading to reduced per-animal impacts at high densities. On the other hand, the suction-pumping mechanism could pull water from deeper in the benthos, potentially oxygenating a greater region of the sediment. This has the potential to increase sediment productivity by increasing the amount of the benthos available for use by aerobic organisms. Bernoulli’s principle is also sensitive to changes in flow velocity, while the suction pumping mechanism is more affected by the frequency of bell pulses. As such, to more accurately predict the ecological implications of the porewater release mediated by *Cassiopea*, we seek to determine to what extent either of these mechanisms are responsible for the porewater release observed in *Cassiopea* sp.

The rate of porewater release by *Cassiopea* is affected by the behavior of the jellyfish in both mechanisms, either by population density in the case of Bernoulli’s principle or by bell pulse rate of individuals in the case of the suction pump mechanism. Since bell pulse rates are temperature-dependent in *Cassiopea*^8^, temperature may also impact porewater and nutrient transfer. *Cassiopea* are found in tropical and subtropical regions, and as such they are cold-sensitive and seasonal changes in water temperature have an effect on their population density and behavior^8^. *Cassiopea* have decreased metabolic rates under cold conditions^8–10^, and low ambient temperatures negatively affect bell diameter and pulse rate^8^. This physiological limitation to mobility has the potential to further impact benthic-pelagic coupling, particularly in the more temperate portions of their range, and for this reason we also investigate the relationship between temperature and behavior in this study.

In this study, we test the proposed hypotheses for the mechanism of *Cassiopea* sp. porewater release and measure bell pulse rates under a variety of in situ and in vitro conditions and conducted a field survey of a *Cassiopea* population density. This information discussed in the context of aiding our understanding of the ecological role of these unique cnidarians.

## Materials and methods

### Housing of *Cassiopea* sp. jellyfish in captivity

*Cassiopea* sp. were collected by hand while wading and snorkeling at our field locations in Lido Key, Florida, and Grassy Key, Florida. Due to the uncertain phylogeny of *Cassiopea* and the visual similarity between species, animals were not identified beyond genus level, although the two species recorded from Florida are *C. andromeda* and *C. frondosa*^11^. These jellyfish were held at the University of South Florida in Tampa, Florida, in a 300 L closed-loop recirculating aquarium system. The salinity in this tank was maintained between 33 and 39‰ with Instant Ocean aquarium salt, and included a substrate of aragonite sand and high-intensity metal halide lighting on a 12:12 light cycle. Water temperature was maintained at ca. 28 °C over the duration of experiments.

### Pressure fluctuations during bell contraction

A 110 L aquarium was filled to a depth of ca. 15 cm with artificial seawater taken from our captive holding tank.A Millar instruments model SPR-524 pressure sensor was fitted through an acrylic platform raised from the bottom so that it was 6.5 cm below the surface of the water, allowing a jellyfish to be placed over the sensor to measure the pressures in the exumbrellar space between the bell and the platform. Pressure readings were collected at 100 Hz using LabChart v.8 software from ADInstruments. The pressure sensor was calibrated using known water depths^12^. Following calibrations, a jellyfish was placed directly over the probe so that the bell completely covered the sensor, and pressure readings were recorded for at least two minutes. Pressure records were made for 18 animals ranging in size from 5.0 to 29.5 cm maximum bell diameter.

**Figure 2:**
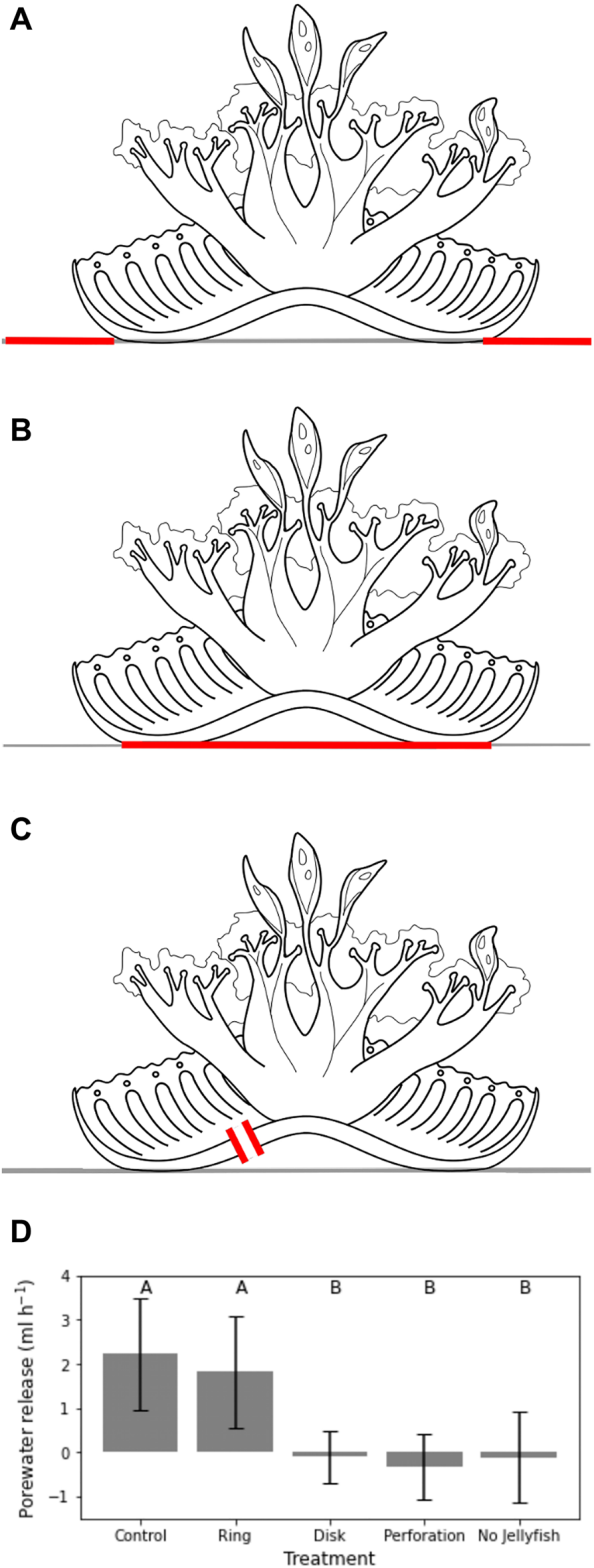
Porewater barriers. Release of porewater was highest in the control group and ring treatments, and absent in both the disk and perforation treatments, as well as the trials without jellyfish. Besides an unaltered control treatment, experimental treatments consisted of (**A**) an impermeable ring surrounding the animal, (**B**) an impermeable disk below the bell, and (**C**) a 6 mm perforation through the bell into the exumbrellar cavity. (**D**) Differences between treatments were found to be significant (one-way Anova, f = 8.61, p < 0.0001) and post-hoc comparisons showed that the two treatments with high porewater release rate (**A**) and low porewater release rates (**B**) were not significantly different within groups, but significantly different between groups (Tukey HSD, α = 0.05). Negative values in low-release treatments (**B**) are likely due to diffusion of fluorescein inadvertently released during experimental setup into the clean cap sand.

Using a custom Python 3 program, pressure data were smoothed with a 0.1 s rolling mean and zeroed to the median of the recording. For five consecutive bell pulses of each animal, we subtracted the average noise from the magnitude of the pressure peaks to isolate the pressure fluctuation associated with each bell pulse. Correlations between pressure and animal size (diameter and surface area) were performed using linear regression. In addition, for 6 animals, after recording the pressure, a 6 mm circular perforation was made through the bell (Fig. 2C), taking care to avoid the stomachs and bell margins. The animals were then processed again, and the differences between healthy and perforated bells tested using a one-tailed paired t-test.

### Effects of barriers on porewater release

**Figure 3:**
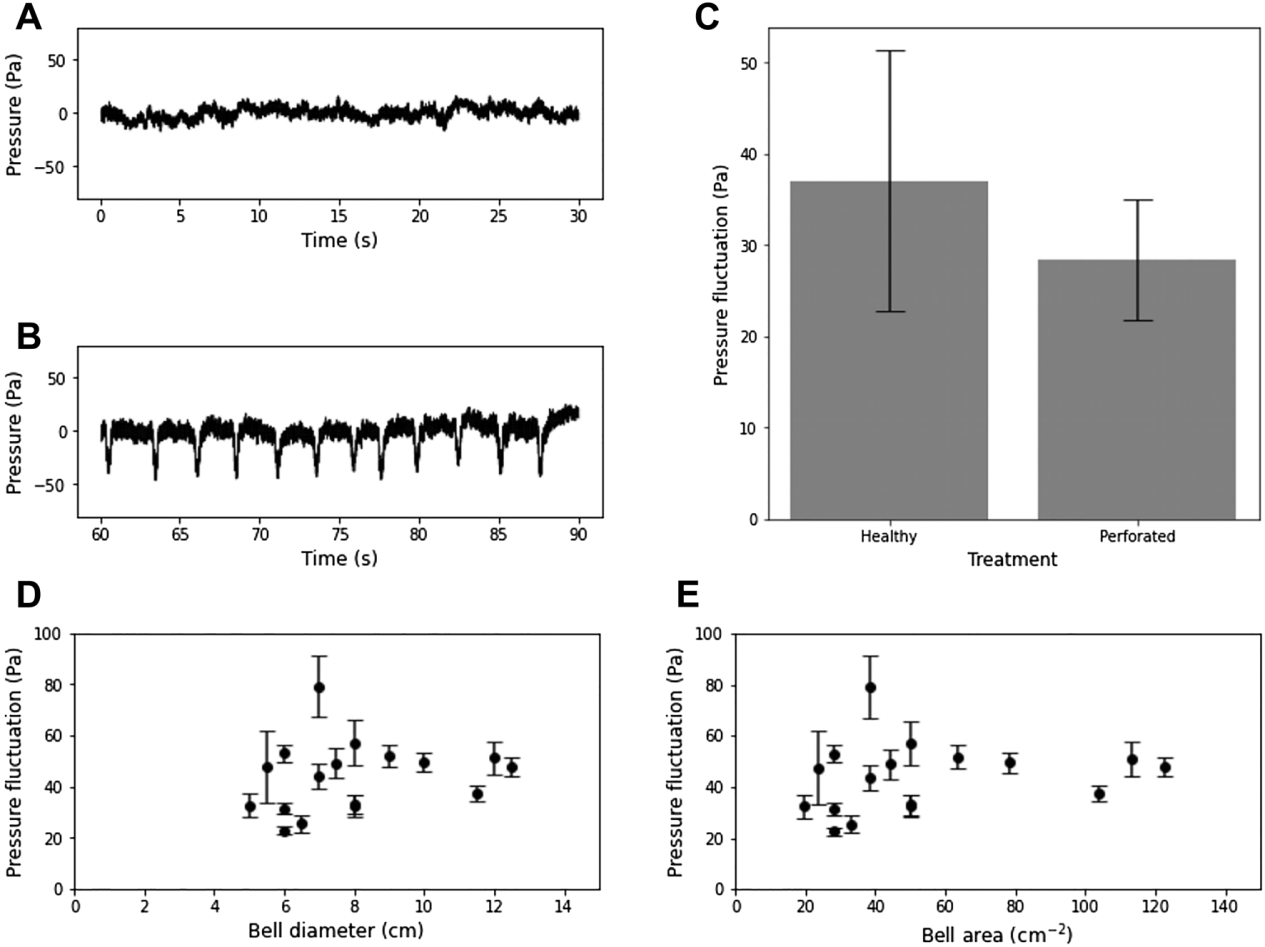
Pumping pressure. Representative pressure data in (**A**) the absence and (**B**) the presence of *Cassiopea* sp. directly over the pressure sensor. Dips in pressure when an animal is present correspond to the initiation of the power stroke and average 43.4 Pa (± 13.6 s.d.). (**C**) The pressure under the bell of jellyfish is reduced when the bell is perforated with a 6 mm perforation. This difference is below the threshold for significance (One-sided Paired t-test, p = 0.07, n = 6, t = 1.81). The mean pressure fluctuation (± s.d.) produced during bell pulsation does not appear to correlate with the (**D**) diameter (n = 17, R^2^ = 0.04) or (**E**) surface area (n = 17, R^2^ = − 0.07) of the bell.

To quantify the rate of interstitial porewater release, we labeled clean silica sand with saturated fluorescein in artificial seawater at 36‰ salinity. Using the approach from^3^, we measured the rate of porewater release over four treatments: (1) A control in which an animal was allowed to settle on the sand for the duration of the experiment; (2) A plastic lining covering the entire sand surface except for a hole of the same diameter as the animal, in which the animal was placed (Fig. 2A); (3) A solid disk of the same diameter as the animal placed between the animal and the sand (Fig. 2B); and (4) A bell perforation trial in which a 6 mm diameter hole was made in the bell before following the same methods as the control (Fig. 2C). Porewater release rates were determined from the ratio of fluorescein concentrations in the sediment to that in the water column at two and three hours after the addition of the animal. One-way analysis of variance was used to determine whether porewater release varied between treatments, and post-hoc Tukey tests identified the specific differences.

### Flow imaging

The flow around healthy *Cassiopea* sp. was imaged using the Particle Image Velocimetry (PIV) methods from^3^, such that both the incurrent and excurrent flow of the feeding current could be observed. The imaging setup consisted of a 45 × 45 × 45 cm aquarium, filled with artificial seawater. The water was seeded using 10 μm reflective hollow glass spheres for particle image velocimetry (PIV). An Edgertronic high-speed camera filming at 50 frames per second provided a field of view ca. 30 × 30 cm. Two 2-W continuous wave DPSS lasers (wavelength = 520 nm), spread through cylindrical lenses to produce narrow light sheets, were staggered one above the other to illuminate a single coronal plane across the entire field of view. One jellyfish at a time (n = 9) was placed on the bottom in the center of the aquarium, such that the laser sheet crossed the center of the animal. After allowing it to settle for about 10 min, 30 s of video were recorded at 50 frames per second. Analysis using the LaVision software package produced a PIV time-average over the 30 s. PIV was processed with interrogation windows between 48 and 64 pixels, at 50% overlap.

### Bell pulse rate and field populations

Bell pulse rates were aggregated from a large number of experiments and field observations dated between 2016 and 2021, for which temperature, salinity, and bell diameter were available. These data represent a total of 330 pulse rate observations. We tested for correlation between bell pulse rate, temperature, salinity, and bell diameter, using multiple linear regression, where diameter was linearized through a semilog transformation.

In order to validate theoretical temperature minima we measured population densities of *Cassiopea* sp. over the winter of 2019–2020. We surveyed a grid of 20 quadrats of 0.25 m^2^ at Lido Key, Florida (27°18′11.8"N 82°33′56.6"W), and recorded jellyfish population densities and bell diameters. Quadrats were placed in 5 m increments along rows every 10 m perpendicular to a 60 m transect line. In addition, to track temperature fluctuations between field excursions, we incorporated daily minimum air temperatures reported by the Sarasota-Bradenton International Airport, 10 km North of the field site.

## Results

### Pressure fluctuations during bell contraction

Pressure fluctuations were expectedly minimal in the absence of an animal (Fig. 3A) and visible pressure minima coincided with the initiation of the power stroke of bell pulsation (Fig. 3B). The magnitude of the pressure fluctuations averaged 43.4 Pa (± 13.6 s.d.) and did not appear to correlate with either animal bell diameter (Fig. 3D, n = 18, R^2^ = 0.002) or surface area (Fig. 3E, n = 18, R^2^ = − 0.003).

Bell perforation was followed by a short period of increased bell pulsation rates and mucus production, but after a brief recovery period there appeared to be no effect on the behavior of the animals. A decrease in the mean magnitude of pressure fluctuations from 37.0 Pa (± 15.6 s.d.) to 28.3 Pa (± 7.2 s.d.) was observed within individual jellyfish following bell perforation (Fig. 3C), and this difference was found to be statistically significant at α = 0.1 (One-sided Paired t-test, n = 6, t = 1.81, p = 0.07).

### Porewater release rates

Porewater liberation rate, as measured through fluorescein concentrations (Fig. 2D) in the presence of healthy *Cassiopea* sp. averaged 2.23 mL h^−1^ (± 1.27 s.d.), while this average was reduced to − 0.11 mL h^−1^ (± 1.024 s.d.) in the absence of *Cassiopea* sp. indicating negligible rates of fluorescein absorption, rather than release, over time. Between all treatments, porewater release varied significantly (one-way Anova, f = 8.61, p = 0.0001). Post-hoc pairwise comparisons (Tukey HSD, α = 0.05) showed that control and ring treatments were not significantly different from each other, and that disk and perforation treatments did not release fluorescein at rates significantly different from trials without jellyfish.

### Flow imaging

**Figure 4:**
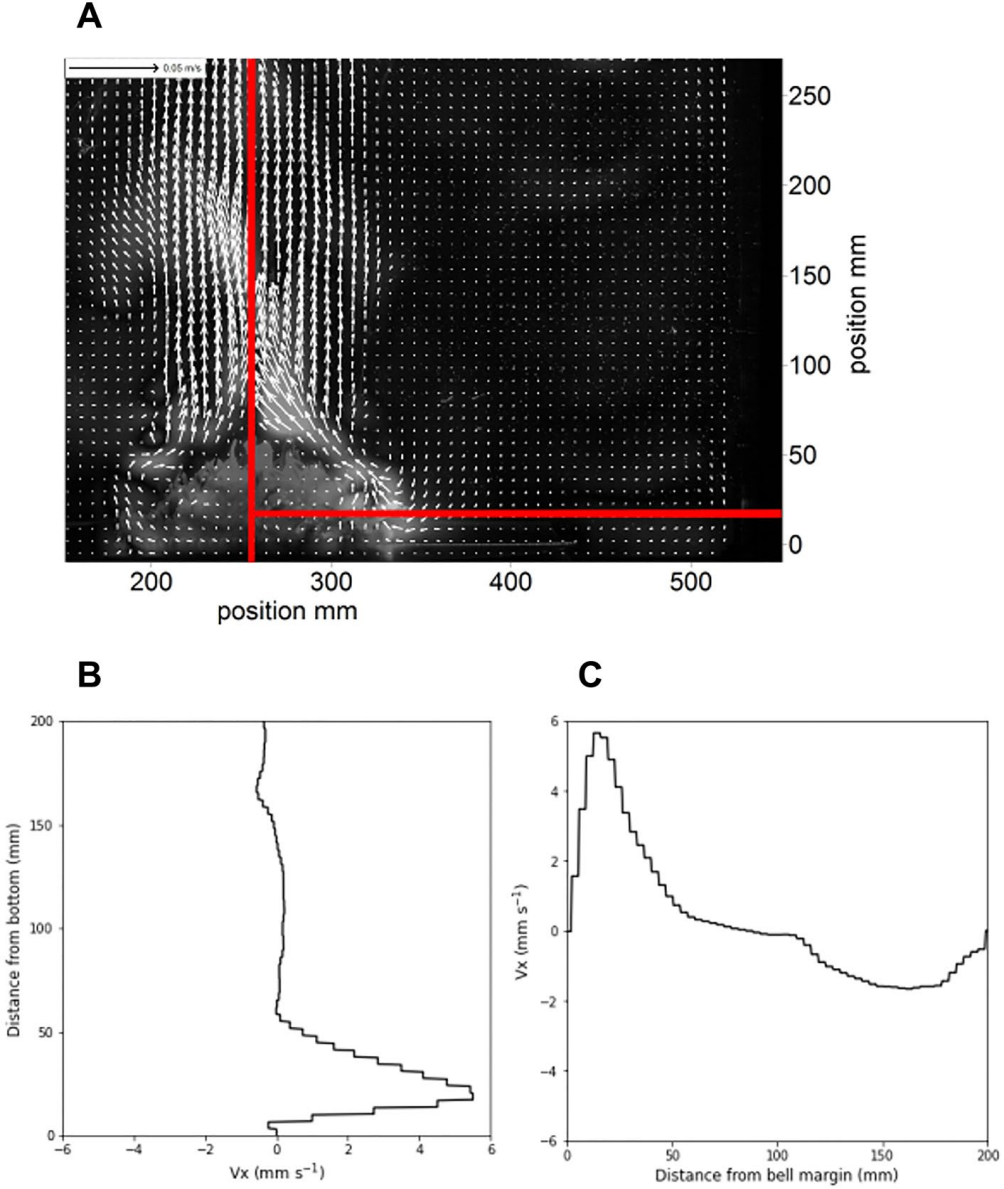
Flow components. The feeding current of *Cassiopea* sp. imaged via Particle Image Velocimetry. (**A**) The shape of the flow shows the slow incurrent flow near the benthos and the more rapid excurrent vertical flow. (**B**) A vertical profile 1 cm to the right of the bell margin of the horizontal component of water velocity shows that a peak velocity of < 6 mm s^−1^ occurs 2.5 cm above the bottom. (**C**) A horizontal profile at 2 cm above the bottom shows that increased horizontal flow is limited to a narrow region near the bell margin, with peak velocities occurring within 2 cm of the bell.

Particle Image Velocimetry (PIV) demonstrates that the incurrent flow is of low velocity and restricted to a narrow region near the bell less than 5 cm from the bottom (Fig. 4). Horizontal flow occurred within 85 mm horizontally from the bell margin. At 47 mm from the bell margin, horizontal velocities first increased to above 1 mm s^−1^ and continued to rise to a peak velocity of 5.5 mm s^−1^ at a distance of 12 mm from the bell margin. After this point, the flow was diverted upward, reducing horizontal velocity.

### Bell pulse rate and field populations

**Figure 5:**
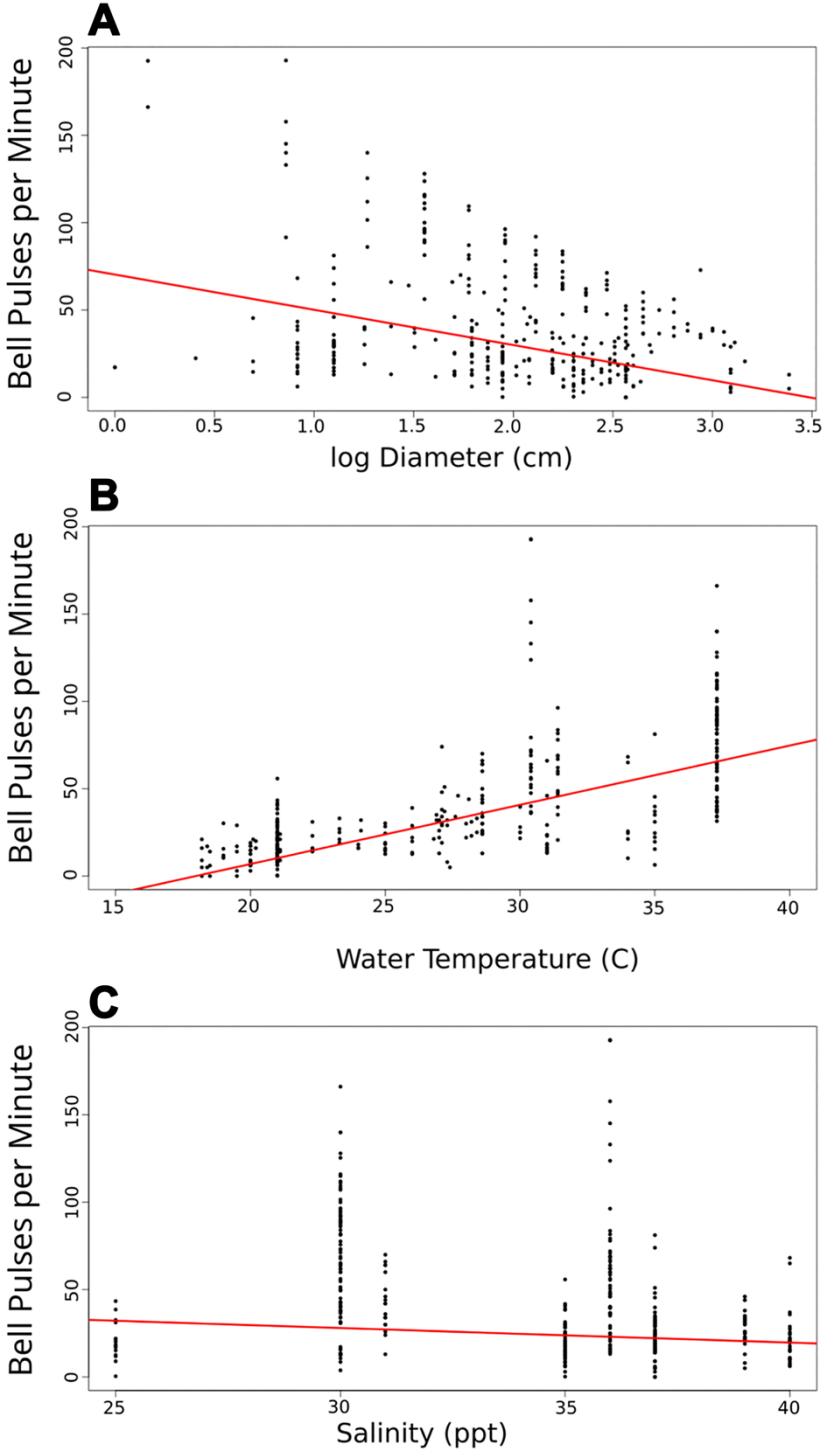
Pulse rate model. A multiple linear regression correlating bell pulsation rate to (**A**) log of jellyfish diameter (R^2^ = 0.34), (**B**) water temperature (R^2^ = 0.42), and (**C**) salinity (R^2^ = 0.07). This model explained a majority of the variation in bell pulse rates (n = 330, R^2^ = 0.57). Pulse rate correlated negatively with the natural log of diameter, positively with temperature, and no correlation was observed with salinity.

**Figure 6:**
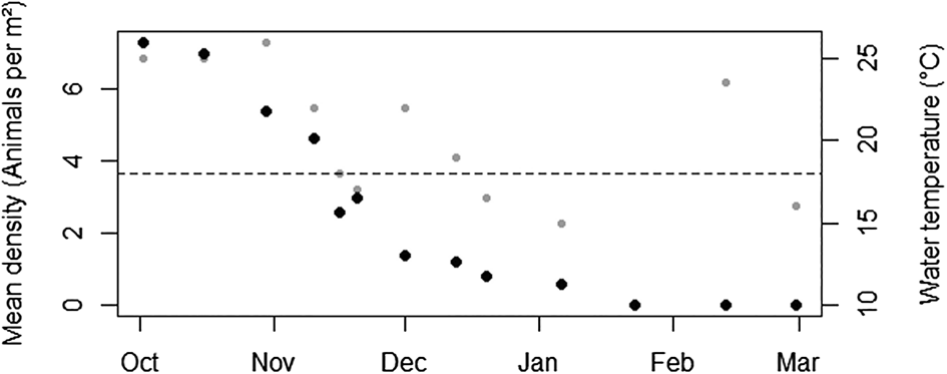
Field population density. Population densities of *Cassiopea* sp. at Lido Key, Florida, through the winter of 2019–2020. Density (black) declines throughout the season, and population decline coincides with low water temperatures (grey). Horizontal dashed line indicates 18 °C water temperature.

Bell pulse rates fit a multiple linear regression with the equation:

1 $$BPR = -20.16\, log(D) + 3.39 T - 0.83 S + 14.58,$$

where BPR is the bell pulse rate in pulses per minute, D is maximum bell diameter (cm), T is temperature (°C), and S is salinity (‰). This regression explains a majority of the variation in bell pulse rate (Fig. 5, n = 330, R^2^ = 0.57). While all independent variables were statistically significant to the model (p < 0.05), bell diameter (R^2^ = 0.34) and water temperature (R^2^ = 0.42) had a much larger effect on BPR than salinity (R^2^ = 0.07) (Fig. 5). Our model predicts that animals with bell diameters of 10 cm would cease pulsing when water temperatures drop to ca. 18 °C. At our field site at Lido Key, Florida, individual bell diameters ranged from 4.4 to 18.7 cm with an average of 9.5 cm. Field populations were in decline when temperatures dropped below 25 °C, but fell rapidly to zero once temperatures dropped below 18 °C (Fig. 6).

## Discussion

The upside-down jellyfish, *Cassiopea* sp. produces several hydrodynamic effects capable of altering the ecosystem which it inhabits. Not only do *Cassiopea* produce feeding currents capable of turning over the water column above them several times per hour^3^, they are also capable of releasing interstitial porewater from the benthos^5^. The rate of porewater release, on the order of mL h^−1^^3^, is capable of increasing water column NH_4_ levels by almost 30% under certain conditions^3^. In this study, we investigated two hypothetical mechanisms for this porewater release, and found that a combination of the morphology of the bell and the pulsing behavior of the jellyfish was responsible for releasing porewater from directly below the bell via a suction-pumping mechanism.

The Bernoulli hypothesis^4^, a low-pressure zone surrounding the animal due to a velocity gradient between the substrate boundary and the incurrent flow of the *Cassiopea* sp. feeding current, predicted porewater release from the substrate surface surrounding the perimeter of the animal. While porewater is entrained from the perimeter of the bell into the feeding current^4^ lateral expulsion of porewater due to the suction pump mechanism would produce a visually similar flow of porewater. A horizontal flow of water does occur near the bottom^1^, but this flow is restricted to a narrow region near the bell and velocities were low compared to the vertical excurrent jet (Fig. 4). To test the effect of Bernoulli’s principle, we measured the effect on porewater release rates of an impermeable ring-shaped barrier surrounding the animal in order to inhibit benthic-pelagic fluid flux other than directly under the animal (Fig. 2A) using labeled fluorescein per the methods of Durieux et al*.*^3^, which were adapted from those of Jantzen et al*.*^5^ (Fig. 2). If the Bernoulli mechanism contributed to porewater liberation this treatment should have reduced the porewater release rate, but the release rates observed were not significantly different from the control treatment (2.23 mL h^−1^ ± 1.27 s.d., Fig. 2D).

The suction pumping hypothesis^5^, a mechanism using the exumbrellar cavity as a suction pump that draws porewater vertically upward beneath the bell and then expels it laterally, would expect to see the majority of porewater released from directly under the bell of *Cassiopea* sp. This mechanism is supported by bell morphology^5^ and the appearance of deep porewater at the benthic surface of the exumbrellar cavity^5^. In our, an impermeable disk was placed underneath the animal to obstruct the flow predicted by the suction pump hypothesis (Fig. 2B). Additionally, we made a 6 mm perforation in the bells of the jellyfish to interfere with the ability to form the sub-ambient pressure in the exumbrellar space necessary for suction pumping to occur (Fig. 2C). Both treatments resulted in a significant decrease in porewater liberation, with flows indistinguishable from the absence of any animal (Fig. 2D), supporting the suction-pumping hypothesis.

Since the suction pumping mechanism requires pressure fluctuations in the exumbrellar space, we also directly measured the water pressure below the jellyfish. The initiation of the power stroke of bell pulsation coincides with a sudden decrease in water pressure in the exumbrellar space (Fig. 3A,B) of a mean magnitude of 43.4 Pa (± 13.6 s.d.). These pressure fluctuations appear to be unaffected by animal size (Fig. 3D,E), although the rate of porewater release is known to scale with bell diameter^3^. Note that the muscles responsible for bell contraction in *Cassiopea* sp. are roughly 2-dimensional sheets^13^ with a thickness of one cell^14^ and therefore the cross-sectional area also does not scale with diameter. Our experiments were performed on smooth acrylic rather than sand, so that the conditions here were optimal for the formation of a tight seal with the bottom. However, the magnitude of this difference is likely to be small, as *Cassiopea sp.* produce copious amounts of mucus, which can compensate for small-scale surface roughness. In addition, the duration of each individual bell pulse is short^1^, so given the fine pore size of a sand or mud substrate, it is unlikely that subambient pressure would have the opportunity to dissipate enough to affect the high suction impulse produced.

While not statistically significant, bell perforation did lead to data suggesting a decrease in exumbrellar pressure fluctuations (Fig. 3C), which could explain the reduction in porewater release observed (Fig. 2C). The fact that some pressure fluctuation was seen despite a complete lack of porewater release suggests that a minimum magnitude of pressure fluctuation might be necessary for suction pumping to occur. Furthermore, the effect may have been reduced by the ability of injured *Cassiopea* to produce copious amounts of mucus, which could have acted to minimize the impact of bell perforation. These parallel lines of reasoning firmly suggest that suction-pumping is, in fact, the dominant mechanism by which *Cassiopea* sp. release porewater.

The suction-pumping mechanism for the release of porewater has broad-ranging ecological implications. Release rates should increase additively with population density, and the rate of bell pulsation should correlate with the rate of porewater liberation. The additive relationship to population density is important, since *Cassiopea* can occur at high densities of up to 100 animals m^−2^^3^. Furthermore, while the Bernoulli mechanism predicted that interstitial water movement would be limited to the upper layers of the benthos, the suction pump mechanism has the potential to release porewater from deeper sediment strata. This deep flushing should expand the oxygen penetration depth downward, affecting factors such as respiration and sediment stability^15^. Given the fact that *Cassiopea* are capable of moving along the substrate^5,16^ this also means that the oxygen penetration depth is likely to fluctuate over time, favoring organisms that are able to adapt their metabolism or are able to relocate themselves^17^.

Given that porewater at the field site in Long Key, Florida, from which the animals in this study were collected, has mean ammonium concentrations of 72 μM, 160 times higher than the surrounding water column^11^, any benthic-pelagic coupling mechanisms in this habitat could alter nitrogen dynamics, especially given the fact that many marine primary producers preferentially take up ammonium, the most reduced state of nitrogen available, as a nitrogen source^18^. *Cassiopea* sp. animal size and population densities are known to correlate with anthropogenic disturbances, and it is suggested that this is due to an increase in nutrient availability in these areas^6^. In addition to prey capture, *Cassiopea* sp. could be supplementing their nitrogen demand through the release of nutrient-rich interstitial porewater, from which *Cassiopea* sp. can directly absorb ammonium and other nutrients such as phosphate and trace metals^5^. In fact, jellyfish presence significantly reduced porewater ammonium levels near the animal^5^, suggesting that nutrient-rich porewater was replaced by down-welling low-nutrient surface water. The observed benthic locomotion of *Cassiopea*^5,16^ may be a mechanism to avoid remaining in locations where they have depleted this nutrient resource^3^. It has been reported that *Cassiopea* sp. affect benthic nutrient transport on a more general level, increasing ammonium uptake and decreasing nitrate uptake of the bottom sediments^19^. Water column nutrient levels also varied significantly between presence and absence of *Cassiopea* sp., and also between light and dark treatments in the presence of *Cassiopea* sp.^20^. The addition of jellyfish increased the efflux of ammonium from the benthos during the dark treatments, but reduced ammonium concentrations in the water column during light treatments^20^. It is entirely possible that absorption of nutrients by *Cassiopea* sp.^5^ in order to meet daytime metabolic demand resulted in the animals reducing water column ammonium concentrations in these experiments^20^.

In addition, *Cassiopea* sp. have been shown to increase spatial heterogeneity of interstitial oxygen and nutrient fluxes^20^, making it comparable to other biogenic processes like bioturbation. Bioturbation typically oxygenates the upper layers of substrate, increasing the nitrification zone^21^, and also increases 3-dimensional heterogeneity of oxygen and nutrient concentrations, allowing for more complex nutrient dynamics^21^. The transport of interstitial porewater from specific regions under individual jellyfish could well produce a similar effect. The porewater release rates can also be compared to that of abiotic processes, such as wind-wave driven flow over sediment wave ripples, which have been shown to liberate porewater at rates of up to 140 L m^−2^ day^−1^, or three orders of magnitude greater than diffusion alone, on shallow, exposed coastlines such as beaches^22^. Environmental mixing would be lower in the sheltered mangrove habitats where *Cassiopea* sp. are found, since at our study site wind wave height was reduced from 5.4 cm in the bay to 0.07 cm in the mangroves^3^. In these coastal habitats, the sediment often acts as a nutrient sink, causing certain nutrients to become limiting to primary producers. Some fringe mangrove forests along coastlines in both Florida and Belize have been shown to be N-limited, for example^23,24^. If these nutrients are then released back into the water column, they potentially increase primary productivity in the system occupied by *Cassiopea* sp. Depending on the system, this could either increase production or cause eutrophication, potentially altering productivity on a local or regional scale, as has been observed when nutrients are released from the benthos by winds^25^ or bioturbation^26^.

The mechanics of suction-pumping also imply that interstitial porewater release rate will correlate with bell pulse rate. Pulse rate correlates with water temperature (Fig. 5B), which would suggest that *Cassiopea* sp. can release greater quantities of nutrient-rich porewater during the summer months. This was confirmed by a recent study on the related species, *Cassiopea medusa* from Lake Macquarie, Australia^8^. By extension, our model suggests that pulsing, and therefore porewater release, should cease entirely below 18ºC. In fact, at our site in Lido Key, population densities of *Cassiopea* sp. declined rapidly once water temperatures dropped this low (Fig. 6). This same temperature of 18 °C was determined independently to be the threshold at which *Cassiopea* sp. polyp feeding was inhibited^10^. As such, it is likely that winter minimum temperatures of 18ºC represent a limiting condition on *Cassiopea* sp. range expansion. Studies on *Cassiopea medusa*, suggested thermal stress and bell degradation at 16 °C^8^. As global climates warm, we can expect both a poleward shift of *Cassiopea* sp. Range^9,27^ and an increase in transport rates of porewater and its associated benthic nutrients throughout this range, leading to increased productivity, and potentially exacerbating eutrophication in some regions.

We determined that a suction-pumping mechanism is responsible for the interstitial porewater release by *Cassiopea*, suggesting that release rates are independent of population density, but affected by pulse rate. The potential role of bell pulse rate was investigated further, and we found correlations between bell pulse rate and both animal size and water temperature. As a result, we expect that porewater liberation would demonstrate seasonal variations, with lower rates during the winter and reaching a maximum during the summer months. *Cassiopea* are able to release nutrient-rich porewater in the shallow quiescent habitats they inhabit, and through their feeding current mix these nutrients throughout the water column. Since this effect varies seasonally, it is likely that further study will show that these jellyfish are responsible for a complex system of nutrient dynamics in their ecosystem.

## Data Availability

All data publicly available at: https://doi.org/10.6084/m9.figshare.20425302.v1.

## Abbreviations

D: Maximum bell diameter (cm)
T: Temperature (°C)
S: Salinity (‰)
PIV: Particle image velocimetry
R^2^: Coefficient of determination
N: Number of measurements
BPR: Bell pulsation rate (pulses per minute)
